# Triglyceride-glucose index as a valuable predictor for aged 65-years and above in critical delirium patients: evidence from a multi-center study

**DOI:** 10.1186/s12877-023-04420-0

**Published:** 2023-10-30

**Authors:** Xiaxuan Huang, Hongtao Cheng, Shiqi Yuan, Yitong Ling, Shanyuan Tan, Yonglan Tang, Chen Niu, Jun Lyu

**Affiliations:** 1 Department of Neurology, The First Affiliated Hospital of Jinan University, Guangzhou, 510630 China; 2 School of Nursing, Jinan University, Guangzhou, 510630 China; 3 Department of Neurology, Guihang Guiyang Hospital, Guiyang, Guizhou 550000 China; 4 Department of Clinical Research, The First Affiliated Hospital of Jinan University, Guangzhou, 510630 China; 5 Guangdong Provincial Key Laboratory of Traditional Chinese Medicine Informatization, Guangzhou, 510630 People’s Republic of China

**Keywords:** Triglyceride glucose index, Insulin resistance, Delirium, Older adults, Critical care

## Abstract

**Background:**

The triglyceride-glucose index (TyG), an established indicator of insulin resistance, is closely correlated with the prognosis of several metabolic disorders. This study aims to investigate the association between the TyG index and the incidence of critical delirium in patients aged 65 years and older.

**Methods:**

We focused on evaluating patients aged 65 years and older diagnosed with critical delirium. Data were obtained from the Medical Information Database for Intensive Care (MIMIC-IV) and the eICU Collaborative Research Database (eICU-CRD). Multivariate logistic regression and restricted cubic spline (RCS) regression were used to determine the relationship between the TyG index and the risk of delirium.

**Results:**

Participants aged 65 years and older were identified from the MIMIC-IV (n = 4,649) and eICU-CRD (n = 1,844) databases. Based on optimal thresholds derived from RCS regression, participants were divided into two cohorts: Q1 (< 8.912), Q2 (≥ 8.912). The logistic regression analysis showed a direct correlation between the TyG index and an increased risk of critical delirium among ICU patients aged 65 and older. These findings were validated in the eICU-CRD dataset, and sensitivity analysis further strengthened our conclusions. In addition, the subgroup analysis revealed certain differences.

**Conclusion:**

This study highlights a clear, independent relationship between the TyG index and the risk of critical delirium in individuals aged 65 years and older, suggesting the importance of the TyG index as a reliable cardio-cerebrovascular metabolic marker for risk assessment and intervention.

**Supplementary Information:**

The online version contains supplementary material available at 10.1186/s12877-023-04420-0.

## Introduction

Delirium, an acute neuropsychiatric syndrome characterized by altered consciousness and cognitive impairment, is a frequently overlooked manifestation of organ dysfunction in older adults with acute medical illness [1–3]. It is associated with an increased risk of adverse clinical outcomes in the short or long term and is most common in intensive care unit (ICU) [4, 5]. The underlying pathophysiology of delirium remains complex and speculative, involving various mechanisms contributing to nerve conduction disorders, neuroinflammation, inadequate brain metabolism, and neurotransmitter imbalances [6, 7]. Delirium in elderly patients typically results from a complex interplay of factors, leading to exacerbated challenges in the ICU setting, such as prolonged hospitalization, increased mortality, and impaired quality of life [8–10]. Therefore, prospective biomarkers associated with delirium need to be explored to help reduce its risk.

Emerging evidence suggests that insulin resistance (IR) is strongly associated with several cerebrovascular diseases and cognitive decline. IR is a metabolic condition characterized by an impaired response of target tissues to insulin, resulting in abnormal glucose and lipid metabolism [11–13]. To assess IR, an index derived from fasting triglyceride and glucose levels, known as the TyG index, has been proposed as a simpler and more stable surrogate measure [14]. Significantly, the TyG index has demonstrated stand-alone predictive ability for conditions such as coronary heart disease, chronic kidney disease, stroke, and carotid atherosclerosis, fueling interest in its possible link to cognitive decline [15–18]. However, the TyG index has not confirmed a definitive association with the onset of ICU delirium in elderly patients, which requires further investigation.

The aim of this study was to investigate the relationship between the risk of delirium in elderly ICU patients and the TyG index and to elucidate the underlying mechanisms, based on a multicenter retrospective design with a large sample size. In addition, we hypothesized that elderly patients with higher TyG index would be at increased risk for adverse outcomes following delirium in ICU.

## Methods

### Data sources

All study data in this study were obtained from and the Medical Information Mart for Intensive Care (MIMIC-IV version 2.0) database [19, 20] and the eICU Collaborative Research Database (eICU-CRD) [21]. The MIMIC-IV database included data from tens of thousands of patients admitted to the ICU at Beth Israel Deakin Medical Center between 2008 and 2019. In contrast, the eICU-CRD included electronic medical records for more than 200,000 patients admitted to ICUs at more than 200 medical centers in 2014 and 2015. Institutional review boards at the Massachusetts Institute of Technology and Beth Israel Deaconess Medical Center approved the study. As such, patient informed consent and ethics approval were waived for this study.

### Cohort selection

**Fig. 1:**
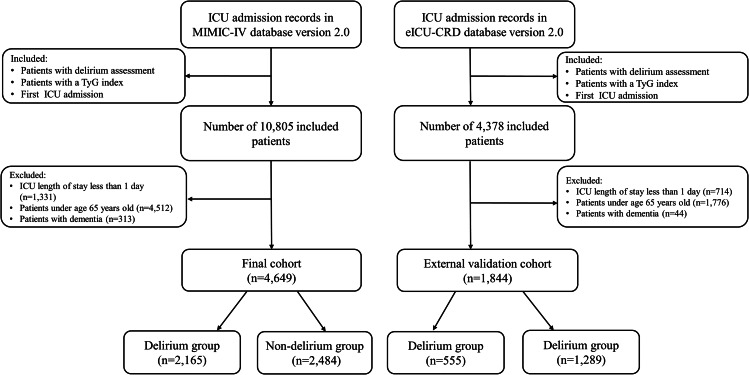
Flowchart illustrating the selection of patients from the MIMIC-IV database and eICU-CRD database Collaborative. Abbreviations: MIMIC-IV, Medical Information Mart for Intensive Care IV; eICU-CRD, eICU Collaborative Research Database; TyG, Triglyceride-glucose

As shown in Fig. 1, we carefully selected our study cohort using strict inclusion and exclusion criteria. Initially, the cohort consisted of patients who had a documented TyG index and underwent delirium assessment during their initial ICU admission. We then excluded patients with an ICU stay of less than 24 h, those under 65 years of age, and those with a diagnosis of dementia, which can easily be confused with delirium. As a result, our study cohort included 4,649 patients, while the external validation cohort included 1,844 patients. We also stratified the study participants into two groups based on delirium status.

### Data collection

In this study, all data was extracted using a structured query language (SQL) server. The data extracted included various demographic characteristics including gender, age, ethnicity. In addition, vital signs such as temperature, heart rate, mean blood pressure, and respiratory rate were documented. Laboratory variables within the first 24 h of ICU admission, including white blood cell (WBC), red blood cell (RBC), platelet count, albumin, blood urea nitrogen (BUN), creatinine, serum sodium, serum potassium, international normalized ratio (INR), triglycerides, and glucose, were also collected. Comorbidities such as sepsis, myocardial infarction, cerebrovascular disease, chronic pulmonary disease, congestive heart failure, diabetes mellitus, renal disease, and malignant cancer were included in the analysis using International Classification of Diseases Ninth Revision (ICD-9) and Tenth Revision (ICD-10) codes. Data on post-admission procedures, including renal replacement therapy (RRT) and mechanical ventilation (MV), as well as length of stay and ICU stay, were also included. The TyG index was calculated from fasting glucose and triglyceride measurements obtained after ICU admission using the formula *In[fasting triglycerides(mg/dl) × fasting blood glucose(mg/dl)/2]* [22, 23].

### Outcomes

The outcome variable of interest in this study was delirium, and the diagnosis of delirium relies on two main assessment tools, the Confusion Assessment Method for the ICU (CAM-ICU) [24] and the Intensive Care Delirium Screening Checklist (ICDSC) [25]. To the best of our knowledge, a bivariate meta-analysis demonstrated that both CAM-ICU and ICDSC had high sensitivity and specificity. The sensitivity for CAM-ICU was 0.84, and the pooled specificity was 0.95. For ICDSC, the pooled sensitivity was 0.83, and the pooled specificity was 0.87 [26]. Although in the MIMIC-IV database, only CAM-ICU assessment results were recorded, both assessment tools are available in the open eICU-CRD database. In addition, it should be noted that a small proportion of delirium patients in the MIMIC-IV database were diagnosed based on the care text diagnostic markers found in the “chartevents” table (itemid = 220,001). The key words for delirium diagnosis included “delirium”, “confusion”, “agitation” and “altered mental status”.

### Statistical analysis

In this study, patients were divided into two groups according to whether they developed delirium while in the ICU. Differences were compared using chi-squared tests for categorical variables and Student’s t-test for continuous variables, with non-normal continuous variables expressed as medians over interquartile ranges.

To assess the association between the TyG index and the risk of ICU delirium, we used univariate and multivariate restricted cubic spline (RCS) regression to evaluate a possible nonlinear association between the TyG index and the risk of delirium in patients aged 65 years and older. We grouped the TyG index based on the cut points obtained from the RCS regression, using the lowest TyG index value as the reference group, and we used logistic regression to assess the risk ratios (ORs) and 95% confidence intervals (CIs) between the continuous variable per 1 unit and the grouped TyG indices on the primary outcome. Given the considerations of multicollinearity and clinical experience, we built three models, of which, model 1 was not adjusted for covariates; in model 2, adjusted for sex, age, laboratory parameters (albumin, serum potassium, serum sodium, creatinine, WBC, RBC, platelet); model 3 was fully adjusted for comorbidities (congestive heart failure, chronic pulmonary disease, myocardial infarction, liver disease, cerebrovascular disease, diabetes, renal disease, hypertension, malignant cancer) based on model 2. To avoid model overflow due to multicollinearity among variables, we also calculated variance inflation factors and excluded variables with variance input factors ≥ 2 (Supplementary Table 1).

To ensure the robustness of our findings, sensitivity analyses were performed in three scenarios. Firstly, patients with an ICU mortality outcome were excluded to minimize the influence of competing death outcomes on the risk of delirium. Secondly, patients with pre-existing diabetes were excluded to specifically examine the risk of delirium in patients without diabetes that is not strongly associated with the TyG index. Sepsis is known to be a major cause of delirium in the elderly, and we further excluded patients with sepsis to reduce the drawback of confounding factors. Lastly, propensity score matching (PSM) [27] was utilized to address any baseline imbalances between both groups of TyG patients and to reduce the potential impact of between-group differences on the models.

In addition, we performed stratified analyses based on several factors, including sex, age (≤ 80 and > 80 years), ethnicity (White and other), sepsis, chronic pulmonary disease, diabetes, congestive heart failure, and hypertension. The consistency of TyG index in predicting delirium was evaluated among subgroups.

Data analysis was performed using R software (version 4.1.3). A two-sided P value of < 0.05 was considered statistically significant.

## Results

### Baseline characteristics

After retrieving data from the MIMIC-IV database and the eICU database, a total of 4,649 patients from MIMIC-IV and 1,844 patients from the eICU were included in this study, following the specified inclusion and exclusion criteria. Among the enrolled patients, the median age was 76 years for the MIMIC-IV database and 75 years for the eICU database. The female population accounted for 46.1% (2,143 patients) in the MIMIC-IV database and 49.2% (907 patients) in the eICU database. The median TyG index were 8.9 (8.5, 9.4) and 8.9 (8.4, 9.3) for the MIMIC-IV and eICU databases, respectively. A total of 2,165 patients with delirium were included in the MIMIC-IV database and a total of 555 patients with delirium were included in the eICU database, as shown in Table 1. Especially in the MIMIC database, patients with delirium had higher WBC, RBC, BUN, creatinine, glucose, and triglyceride levels, and were more likely to have cerebrovascular disease, sepsis, and adverse outcomes (P < 0.001).

**Table 1:** The Baseline clinical characteristics of MIMIC-IV and eICU-CRD patients

Categories	MIMIV-IV				eICU-CRD			
	Overall	Non-delirium	Delirium	*P* value	Overall	Non-delirium	Delirium	*P* value
Total	4,649	2,484	2,165		1,844	1,289	555	
**Demographic**								
Age, years,	76 (70,83)	76 (70,83)	76 (70,83)	0.911	75 (70,82)	75 (69,81)	76 (70,82)	0.036
Sex, n(%)				0.812				0.999
Male	2,506 (53.9)	1,343 (54.1)	1,163 (53.7)		937 (50.8)	655 (50.8)	282 (50.8)	
Female	2,143 (46.1)	1,141 (45.9)	1,002 (46.3)		907 (49.2)	634 (49.2)	273 (49.2)	
Ethnicity, n(%)				< 0.001				0.265
White	3,168 (68.1)	1,795 (72.3)	1,373 (63.4)		1,394 (75.6)	965 (74.9)	429 (77.3)	
Other	1,481 (31.9)	689 (27.7)	792 (36.6)		450 (24.4)	324 (25.1)	126 (22.7)	
**Vital signs**								
Temperature, °C	36.7 (36.4,37.0)	36.7 (36.4,37)	36.8 (36.5,37.1)	< 0.001	36.4 (36.2,36.7)	36.4 (36.2,36.7)	36.4 (36.1,36.7)	0.821
Heart rate, beats/minute	85 (73,100)	84 (72,98)	86 (74,102)	< 0.001	84 (70,100)	82 (69,98)	86 (74,102.5)	< 0.001
Mean blood pressure, mmHg	84 (72,97)	83 (71,96)	86 (74,99)	< 0.001	67 (53,131)	66 (53,128)	72 (51,137)	0.186
Respiratory rate, beats/minute	19 (16,23)	18 (16,23)	20 (16,24)	< 0.001	19 (16,23)	19 (16,23)	19 (16,23)	0.72
**Laboratory tests**								
Albumin, g/dL	3.4 (2.8,3.9)	3.5 (2.9,3.9)	3.3 (2.8,3.8)	< 0.001	3 (2.5,3.4)	3 (2.6,3.4)	2.9 (2.4,3.3)	< 0.001
BUN, mg/dL	22 (16,36)	21 (16,34)	23 (16,37)	< 0.001	21 (15,33)	21 (15,31)	23 (15,37)	0.005
Creatinine, mg/dL	1.1 (0.8,1.5)	1 (0.8,1.5)	1.1 (0.8,1.6)	0.037	1.1 (0.8,1.6)	1 (0.8,1.5)	1.1 (0.8,1.7)	0.027
Serum sodium, mEq/L	139 (136,141)	139 (136,141)	139 (136,142)	0.206	139 (136,141)	139 (136,141)	139 (136,142)	0.01
Serum potassium, mEq/L	4.1 (3.8,4.6)	4.2 (3.8,4.5)	4.1 (3.8,4.6)	0.677	4.1 (3.7,4.5)	4.1 (3.7,4.5)	4 (3.7,4.6)	0.564
INR	1.2 (1.1,1.5)	1.2 (1.1,1.5)	1.2 (1.1,1.5)	0.116	1.1 (1,1.3)	1.1 (1,1.3)	1.2 (1.1,1.4)	< 0.001
WBC, 10^9^/L	10.6 (7.7,14.7)	10.5 (7.7,14.4)	10.8 (7.7,15.1)	0.057	10.3 (7.7,14.3)	10 (7.4,13.8)	11.6 (8.4,16)	< 0.001
RBC, 10^9^/L	3.7 (3.1,4.2)	3.6 (3.1,4.2)	3.7 (3.1,4.3)	0.358	3.8 (0.7)	3.9 (0.7)	3.8 (0.8)	0.007
Platelet, 10^9^/L	204 (150,267)	205 (152,269.2)	201 (150,265)	0.119	197 (152,250)	199 (153,246)	194 (149,260.5)	0.933
Triglycerides, mg/dL	109 (79,156)	106 (77,149)	113 (81,165)	< 0.001	102 (76,145)	101 (76,142)	105 (75.5,157)	0.145
Glucose, mg/dL	132 (106,171)	129.5 (105,167)	135 (108,176)	< 0.001	128 (106,168)	126 (103,165)	135 (112,176)	< 0.001
TyG index	8.9 (8.5,9.4)	8.9 (8.4,9.3)	9 (8.5,9.5)	< 0.001	8.9 (8.4,9.3)	8.8 (8.4,9.2)	8.9 (8.5,9.4)	0.003
**Comorbidities**								
Myocardial infarction, n(%)				< 0.001				< 0.001
No	3,390 (72.9)	1,716 (69.1)	1,674 (77.3)		1,261 (68.4)	832 (64.5)	429 (77.3)	
Yes	1,259 (27.1)	768 (30.9)	491 (22.7)		583 (31.6)	457 (35.5)	126 (22.7)	
Cerebrovascular disease, n(%)				< 0.001				< 0.001
No	2,770 (59.6)	1,651 (66.5)	1,119 (51.7)		1,253 (68)	925 (71.8)	328 (59.1)	
Yes	1,879 (40.4)	833 (33.5)	1,046 (48.3)		591 (32)	364 (28.2)	227 (40.9)	
Chronic pulmonary disease, n(%)				0.363				0.945
No	3,356 (72.2)	1,807 (72.7)	1,549 (71.5)		1,477 (80.1)	1,033 (80.1)	444 (80)	
Yes	1,293 (27.8)	677 (27.3)	616 (28.5)		367 (19.9)	256 (19.9)	111 (20)	
Diabetes, n(%)				0.025				0.343
No	3,058 (65.8)	1,670 (67.2)	1,388 (64.1)		1,267 (68.7)	877 (68)	390 (70.3)	
Yes	1,591 (34.2)	814 (32.8)	777 (35.9)		577 (31.3)	412 (32)	165 (29.7)	
Renal disease, n(%)				0.02				0.887
No	3,446 (74.1)	1,876 (75.5)	1,570 (72.5)		1,555 (84.3)	1,088 (84.4)	467 (84.1)	
Yes	1,203 (25.9)	608 (24.5)	595 (27.5)		289 (15.7)	201 (15.6)	88 (15.9)	
Malignant cancer, n(%)				0.614				0.264
No	3,966 (85.3)	2,113 (85.1)	1,853 (85.6)		1,493 (81)	1,035 (80.3)	458 (82.5)	
Yes	683 (14.7)	371 (14.9)	312 (14.4)		351 (19)	254 (19.7)	97 (17.5)	
Liver disease, n(%)				< 0.001				0.432
No	4,200 (90.3)	2,289 (92.1)	1,911 (88.3)		1,818 (98.6)	1,269 (98.4)	549 (98.9)	
Yes	499 (9.7)	195 (7.9)	254 (11.7)		26 (1.4)	20 (1.6)	6 (1.1)	
Hypertension, n(%)				0.018				0.761
No	2,310 (49.7)	1,194 (48.1)	1,116 (51.5)		544 (29.5)	383 (29.7)	161 (29)	
Yes	2,339 (50.3)	1,290 (51.9)	1,049 (48.5)		1,300 (70.5)	906 (70.3)	394 (71)	
Congestive heart failure, n(%)				0.266				0.663
No	2,978 (64.1)	1,573 (63.3)	1,405 (64.9)		1,420 (77)	989 (76.7)	431 (77.7)	
Yes	1,671 (35.9)	911 (36.7)	760 (35.1)		424 (23)	300 (23.3)	124 (22.3)	
Sepsis, n(%)				< 0.001				< 0.001
No	3,639 (78.3)	2,090 (84.1)	1,549 (71.5)		1,646 (89.3)	1,181 (91.6)	465 (83.8)	
Yes	1,010 (21.7)	394 (15.9)	616 (28.5)		198 (10.7)	108 (8.4)	90 (16.2)	
**Events**								
RRT treatment, n(%)				< 0.001				0.013
No	4,210 (90.6)	2,326 (93.6)	1,884 (87)		1,764 (95.7)	1,243 (96.4)	521 (93.9)	
Yes	439 (9.4)	158 (6.4)	281 (13)		80 (4.3)	46 (3.6)	34 (6.1)	
MV, n(%)				< 0.001				< 0.001
No	2,412 (51.9)	1,594 (64.2)	818 (37.8)		984 (53.4)	758 (58.8)	226 (40.7)	
Yes	2,237 (48.1)	890 (35.8)	1,347 (62.2)		860 (46.6)	531 (41.2)	329 (59.3)	
ICU death, n(%)				< 0.001				< 0.001
Survivor	4,138 (89.0)	2,322 (93.5)	1,816 (83.9)		1,747 (94.7)	1,247 (96.7)	500 (90.1)	
Non-survivor	511 (11.0)	162 (6.5)	349 (16.1)		97 (5.3)	42 (3.3)	55 (9.9)	

Abbreviations: MIMIC-IV, Medical Information Mart for Intensive Care IV; eICU-CRD, eICU Collaborative Research Database; RRT, Renal replacement therapy; MV, Mechanical ventilation; TyG, Triglyceride-glucose; ICU, Intensive care unit.

### Association of TyG index with risk of delirium

**Fig. 2:**
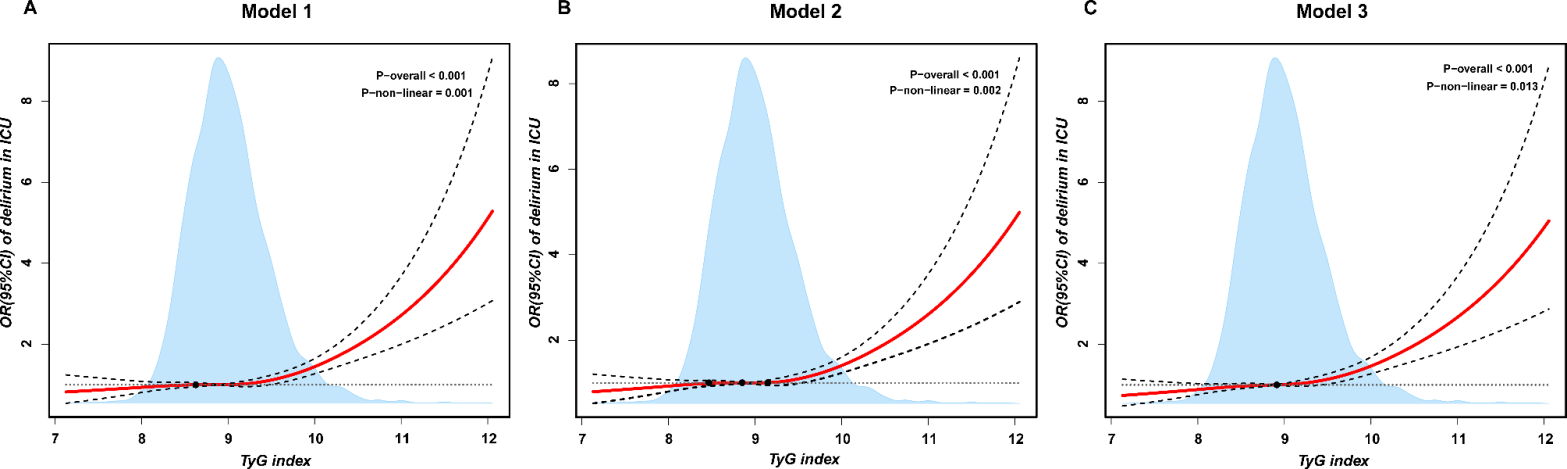
The potential nonlinear relationship between TyG index level and the risk of delirium in elderly ICU patients by restricted cubic spline regression. The red and dashed lines represent the estimated OR and 95% CIs, respectively. **(A)** Model 1 was an unadjusted model; **(B)** Model 2 was adjusted for sex, age, laboratory parameters (albumin, serum potassium, serum sodium, creatinine, WBC, RBC, platelet); **(C)** Model 3 was adjusted for comorbidities (congestive heart failure, sepsis, chronic pulmonary disease, myocardial infarction, liver disease, cerebrovascular disease, diabetes, renal disease, hypertension, malignant cancer) on the basis of Model 2. The final fully adjusted restricted cubic spline regression showed that the cut value of TyG index was 8.912. Abbreviations: TyG, Triglyceride-glucose

We utilized restricted cubic splines to analyze the continuous relationship between the TyG index and the incidence of delirium in ICU. In the fully adjusted multivariate RCS model, our findings indicated a non-linear association between the TyG index and the risk of ICU delirium (P-nonlinear < 0.001, P-overall = 0.013). We observed that the risk of ICU delirium increased when the TyG index larger than 8.912 **(**Fig. 2**)**. Based on the restricted cubic spline analysis, we defined two categories of patients: Q1 (< 8.912) and Q2 (≥ 8.912). Multivariate logistic regression analysis, with Q1 (< 8.912) as the reference group, showed that an elevated TyG index was associated with a higher risk of delirium (OR = 1.312, 95% CI: 1.157–1.488, P < 0.001). These findings were validated in the eICU-CRD dataset, consistently indicating that among ICU patients aged 65 and older, an elevated TyG index was positively correlated with increased delirium risk (OR = 1.259, 95% CI: 1.009–1.570, P = 0.041), especially in those with a TyG index of ≥ 8.912 (Table 2).

**Table 2:** The association between various TyG index groups and risk of delirium in ICU patients aged 65 and above

	Model 1		Model 2		Model 3	
	OR(95%CI)	*P*-value	OR(95%CI)	*P*-value	OR(95%CI)	*P*-value
**MIMIC-IV**						
TyG index^*^	1.294 (1.195–1.404)	< 0.001	1.278 (1.178–1.387)	< 0.001	1.333 (1.221–1.458)	< 0.001
TyG^※^						
Q1(< 8.912)	(Reference)		(Reference)		(Reference)	
Q2(≥ 8.912)	1.313 (1.159–1.489)	< 0.001	1.245 (1.108–1.399)	< 0.001	1.312 (1.157–1.488)	< 0.001
**eICU-CRD**						
TyG index^*^	1.297 (1.122-1.500)	< 0.001	1.258 (1.079–1.468)	0.003	1.374 (1.163–1.625)	< 0.001
TyG^※^						
Q1(< 8.912)	(Reference)		(Reference)		(Reference)	
Q2(≥ 8.912)	1.232 (1.010–1.504)	0.040	1.149 (0.933–1.413)	0.190	1.259 (1.009–1.570)	0.041

**Notes**: *Stands for TyG index were continuous variable per 1 unit. ※Stands for the TyG continuous variables were divided into two groups based on the cut values obtained from the multivariate RCS regression analysis, with the lowest group used as the reference group. Model 1: unadjusted model; Model 2: adjusted for sex, age, laboratory parameters (albumin, serum potassium, serum sodium, creatinine, WBC, RBC, platelet); Model 3: adjusted for sex, age, laboratory parameters (albumin, serum potassium, serum sodium, creatinine, WBC, RBC, platelet) and comorbidities (congestive heart failure, chronic pulmonary disease, sepsis, myocardial infarction, liver disease, cerebrovascular disease, diabetes, renal disease, hypertension, malignant cancer);

Abbreviations: TyG, Triglyceride-glucose; OR, odds ratio; CI, confidence interval.

### Sensitivity analysis of TyG index and the risk of delirium

The sensitivity analyses conducted in this study corroborated the robustness of the primary findings. Firstly, the exclusion of patients who died in the ICU did not significantly attenuate the statistical association between the TyG index and delirium. Secondly, even after excluding patients with sepsis, the relationship between the TyG index and the risk of delirium remained robust. Moreover, even after excluding patients with diabetes, which correlates with the TyG index, a significant association between the TyG index and the risk of delirium remained in patients without a history of the condition (Supplementary Tables 2–4). Lastly, PSM was employed to minimize discrepancies between the groups, The observed results were consistent with the primary analysis, indicating that in ICU patients aged 65 and above, a higher TyG index is associated with an increased risk of delirium (Supplementary Tables 5–6).

### Subgroup analysis

**Fig. 3:**
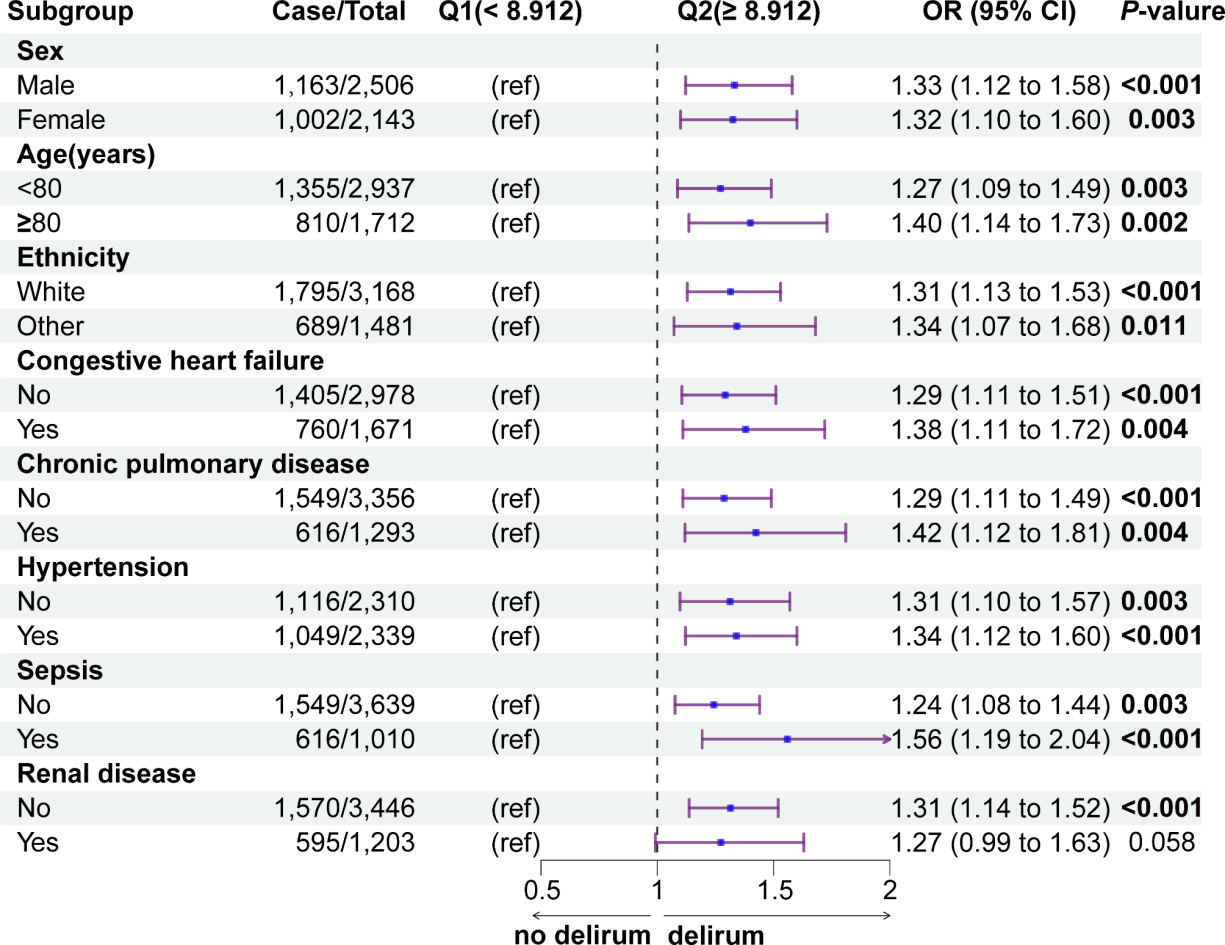
Subgroup analysis for the association of TyG index with the risk of delirium. Abbreviations: TyG, Triglyceride-gluco**se**; OR, odds ratio; CI, confidence interval

Evaluating the association between the TyG index and the risk of delirium amongst geriatric patients in critical care, while taking into account variances such as sex, age, ethnicity, along with comorbidities like congestive heart failure, chronic pulmonary disease, hypertension, sepsis and renal disease, revealed intriguing outcomes. We observed significant differences in the subgroups of white males aged over 80 with comorbidities of congestive heart failure, chronic pulmonary disease, and sepsis. Within these subgroups, a substantial increase in the risk of delirium was evident, when the TyG index values larger than 8.912 (Fig. 3, Supplementary Table 7).

### Further analysis

Our study also found that individuals who succumbed to delirium had a propensity for adverse prognostic outcomes as determined by Kaplan-Meier survival analyses at various follow-up periods including 30 days, 90 days, and 360 days. There was a statistically significant difference in mortality between the two groups (log-rank test: all P < 0.05). It’s worth noting that these results were even more pronounced at the shorter, 30 days follow-up (HR = 2.134, Supplementary Fig. 1).

## Discussion

This study demonstrated a definitive association between the TyG index and the risk of delirium in geriatric critically ill patients. Drawing from the MIMIC-IV and eICU-CRD databases, the study’s conclusions were strengthened by its large sample size and diverse representation. Notably, this was the first multicenter study to correlate the TyG index with delirium susceptibility in elderly ICU patients. It posited that an elevated TyG index correlates with an increased risk of delirium within a certain range for ICU patients aged 65 years and older.

As far as we know, insulin is a peptide hormone mainly secreted by beta cells of the pancreas, which is essential for the regulation of glucose metabolism in peripheral tissues. This hormone plays a special role in the central nervous system (CNS), where each cell type expresses insulin receptors [28]. These receptors are diffusely distributed throughout the brain, establishing insulin signaling pathways that influence brain plasticity and contribute to neurodegenerative processes. It’s noteworthy that insulin’s central influence extends to the hippocampus, entorhinal cortex, and frontal cortex, regions that are integral to the brain’s cognitive functions. In addition, insulin confers protection against the Aβ protein, thereby influencing lipid metabolism and proteases [29, 30]. Consequently, abnormalities in insulin function may precipitate neurodegeneration or cognitive decline. IR, characterized by reduced sensitivity of the body’s target tissues and cells to insulin, rendering them unable to function normally, has been proposed as a potential culprit for these deleterious effects [31]. Prior research indicated that the TyG index, derived from fasting glucose and triglyceride levels, is proposed as a straightforward surrogate marker of IR, predicated on the notion that insulin’s impact on lipolysis is somewhat less pronounced than on glucose metabolism [32, 33]. Compared to the HOMA-IR (Homeostasis model assessment of IR) index, the TyG index showed superior predictive ability for IR as determined by the hyperglycemic clamp test [34]. This easily measurable index is more amenable to large-scale population studies and was used in this study to test the hypothesis of a possible association in patients with delirium.

Up to now, no definitive studies have established a link between IR and delirium, and only some studies have focused on the progression between IR and prevention of postoperative delirium in patients from a therapeutic perspective [11]. some clinical trials of IR in the treatment of postoperative delirium in elderly patients have been published [35, 36]. A clinical trial study determined the safety and effectiveness of IR in preventing delirium after 6 months of IR treatment in patients with postoperative delirium, which indirectly supports the hypothesis of this study that the TyG index, a marker of IR, predicts the incidence of delirium [37]. In addition, the etiology of delirium and dementia has long been suspected to have significant overlap, and a number of studies have confirmed the potential association of IR with cognitive decline and dementia. A retrospective cohort study from the US National Health Information Database published in 2021 found that TyG index as a marker of IR can independently but small predict the development of dementia [32]. Meanwhile, Kai Wang et al. proposed an association between increased TyG index and a higher risk of cognitive decline in men [38]. From a neuropathological perspective, dementia and delirium are closely related, and based on this background, these findings above potentially bridge the link between IR and delirium. The present study was the first to examine the association between the TyG index and the risk of developing delirium in ICU patients aged 65 years and older and found that an elevated TyG index was a strong independent predictor of an increased risk of delirium in ICU patients aged 65 years and older. This association persisted after adjustment for several clinical and laboratory variables. The present study differs from previous studies in that [18], first, we used large multicenter data, MIMIC-IV and eICU-CRD covering multiple centers, allowing for better confidence in the findings. Second, we performed sensitivity analyses using PSM analysis and excluding patients with a diagnosis of diabetes and adverse outcomes to ensure the robustness of the results. Third, we performed the subgroup analysis and found that significant differences in the subgroups of white males aged over 80 with comorbidities of congestive heart failure, chronic pulmonary disease, and sepsis. We also performed an additional analysis of the adverse prognosis of delirium and found that the increased risk of delirium with a high TyG index showed an incremental prediction of the rate of death.

While the exact pathophysiological mechanisms linking the TyG index to delirium remain unclear, the prevailing hypothesis suggests an underlying IR. However, the possibility that this association is mediated by latent diabetes cannot be completely ruled out. Several lines of evidence support this theory. Firstly, extensive animal studies suggest that tau proteins, which are regulated by insulin signaling in the brain, play a central role in the central nervous system, particularly in the hippocampal response to insulin [39, 40]. These changes are exacerbated by the effects of IRS-1 and phosphatase and tension homolog (PTEN) [41]. IR, characterized by a diminished response to insulin from target tissues, plays an important role in neurodegenerative diseases that affect neuronal metabolism. IR could potentially increase tau hyperphosphorylation by inhibiting PI3-K/AKT and enhancing GSK3-β activation, while an imbalance in tau protein phosphorylation is a pathological indicator of cognitive decline. Therefore, dysregulation of insulin signaling pathways may trigger cognitive impairments such as delirium [42]. Secondly, the mechanisms linking IR and neurodegenerative diseases have been explored previously. Abnormal accumulation of Aβ protein has emerged as a new theory in the pathogenesis of psychosis. Insulin inhibits the formation of Aβ fibers and promotes the internalization of Aβ oligomers, thereby limiting their neuronal binding and protecting synapses from Aβ oligomers [43]. With the onset of IR in the brain, degradation and clearance of Aβ are impeded, and abnormal Aβ deposition stimulates glial cells to release a variety of inflammatory factors and generate oxygen free radicals that induce oxidative stress and activate apoptosis [44]. IR may also induce impairment of macrophage endothelial function and exacerbate arterial atherosclerosis and dyslipidemia, all of which could potentially contribute to the development of delirium. Some clinical studies have provided evidence to support this notion [45]. Thirdly, a meta-analysis has shown that patients with type 2 diabetes are more likely to develop Alzheimer’s disease (AD). In type 2 diabetes, IR leads to a high risk of Aβ protein deposition and tau pathology leading to AD symptoms [46, 47]. Importantly, our research findings have practical implications, especially for the early clinical prediction of delirium in critically ill elderly patients, offering support for early interventions to mitigate the risk of adverse outcomes induced by IR. Our research provides new perspectives on the relationship between delirium and IR in ICU patients aged 65 years and older. However, we cannot conclusively establish a causal relationship between a high TyG index and the subsequent presence of delirium.

There are several limitations of this study. First, the retrospective design of our study constrained our ability to capture detailed and dynamic clinical parameters, such as the duration and recovery patterns of delirium. Understanding the association between the TyG index and the course of recovery from delirium in elderly patients is of potential clinical significance. Consequently, prospective studies are needed in the future to further explore this aspect, this will contribute to a more comprehensive understanding of the role of the TyG index in managing delirium within critical care settings. Second, owing to the inherent limitations of our multi-center study design, this research was restricted to variables consistently available in the MIMIC-IV and eICU-CRD. Despite our utmost efforts to adjust for available variables that might affect delirium outcomes in accordance with the clinical context and applying PSM to observable biases, there still exists the possibility of data bias affecting the results due to unincorporated covariates, such as APACHE and SOFA severity of illness scores for critical diseases. Third, although our findings were externally validated, the majority of our patient data were from the United States, which may limit the generalizability of our results to patients aged 65 years and older in other geographic regions. Therefore, caution should be exercised when extrapolating these findings to other population cohorts. Fourth, delirium manifests in different subtypes, including hyperactive, hypoactive, and mixed. Our study did not address these subtypes, which may limit our comprehensive understanding and management of delirium. Fifthly, given that our study was a retrospective analysis from an observational study, it primarily established a correlation between the TyG index and delirium, without effectively substantiating a causal relationship. Subsequent studies are indispensable to establish causality and to observe the dynamics of TyG index over time, thereby affirming its positive prognostic significance in critically ill patients. Finally, our study focused primarily on patients aged 65 years and older in the ICU, and it is uncertain whether these findings can be extrapolated to older patients in general wards or nursing homes. Future research should aim to expand sample sources to include a wider range of regions and patient types to improve the generalizability of the findings.

## Conclusions

In conclusion, the increased IR was independently and positively correlated with an increased risk of critical delirium in patients aged 65 years and older. This finding suggests the potential of the TyG index as an effective cardio-cerebrovascular metabolic marker for risk classification and management in a high-risk geriatric population. Future research should aim to investigate the clinical significance of the dynamic changes in the TyG index under different clinical conditions.

### Electronic supplementary material

Below is the link to the electronic supplementary material.

Supplementary Material 1

## Data Availability

The data were available on the MIMIC-IV website at https://mimic.physionet.org/ and eICU-CRD at https://eicu-crd.mit.edu/. The data in this article can be reasonably applied to the corresponding author.

## Abbreviations

TyG: Triglyceride-glucose
ICU: Intensive care unit
eICU-CRD: eICU Collaborative Research Database
MIMIC-IV: Medical Information Mart for Intensive Care IV
IR: Insulin resistance
FBG: Fasting blood glucose
WBC: White blood cell
ICD: International Classification of Diseases
LOS: Length of stay
IQR: Interquartile range
VIF: Variance inflation factor
OR: Odds ratio
CI: Confidence interval
HR: Hazard ratio
RCS: Restricted cubic spline
PSM: Propensity score matching
HOMA-IR: Homeostasis model assessment of IR
MI: Myocardial infarction
RRT: Renal replacement therapy
MV: Mechanical ventilation
CAM-ICU: Confusion Assessment Method in the Intensive Care Unit
ICDSC: Intensive Care Unit Delirium Screening Checklist
