# Supplementary material for: Triglyceride-glucose index as a valuable predictor for aged 65-years and above in critical delirium patients: evidence from a multi-center study
Source: BMC Geriatr. 2023 Oct 30;23:701. doi: 10.1186/s12877-023-04420-0 (PMC10617052; doi:10.1186/s12877-023-04420-0)

**Supplementary Materials – Content**

**[Supplementary Table 1. The variance inflation factor for all covariates of fully adjusted model. 1](#_Toc146238748)**

**[Supplementary Table 2. The association between various TyG index groups and risk of delirium in the ICU after excluding the in-ICU outcome was death (N=4,138). 3](#_Toc146238749)**

**[Supplementary Table 3. The association between various TyG index groups and risk of delirium in the ICU after excluding patients with sepsis (N=3,639). 4](#_Toc146238750)**

**[Supplementary Table 4. The association between various TyG index groups and risk of delirium in the ICU after excluding patients with diabetes (N=3,058). 5](#_Toc146238751)**

**[Supplementary Table 5. The association between TyG index groups and risk of delirium in the ICU after propensity score matching (N=3,494). 6](#_Toc146238752)**

**[Supplementary Table 6. The baseline clinical characteristics of the patients after propensity score matching in both TyG groups were analyzed. 7](#_Toc146238753)**

**[Supplementary Table 7. Subgroup analyses for the association of TyG index with the risk of delirium in ICU patients aged 65 and above. 9](#_Toc146238754)**

**[Supplementary Figure 1. Kaplan-Meier analysis showed the cumulative incidence of mortality at 30 days, 90 days, and 360 days after delirium in ICU patients aged 65 and above with TyG index. 10](#_Toc146238755)**

# Supplementary Table 1. The variance inflation factor for all covariates of fully adjusted model.

| **Variable** | **VIF** |
| --- | --- |
| Sex | 1.076033 |
| Age | 1.004415 |
| Albumin, g/dL | 1.332620 |
| Serum sodium, mEq/L | 1.038949 |
| Serum potassium, mEq/L | 1.107668 |
| Creatinine, mg/dL | 1.412066 |
| WBC, 10^9^/L | 1.055314 |
| RBC, 10^9^/L | 1.227770 |
| Platelet, 10^9^/L | 1.091687 |
| Chronic pulmonary disease | 1.050201 |
| Liver disease | 1.084190 |
| Cerebrovascular disease | 1.301132 |
| Renal disease | 1.854796 |
| Hypertension | 1.616361 |
| Malignant cancer | 1.089775 |
| Diabetes | 1.160272 |
| Myocardial infarct | 1.100168 |
| Sepsis | 1.223566 |
| Congestive heart failure | 1.233313 |

**Abbreviations:** VIF, Variance inflation factor; WBC, White cell count; RBC, Red cell count

# Supplementary Table 2. The association between various TyG index groups and risk of delirium in the ICU after excluding the in-ICU outcome was death (N=4,138).

|  | **Model 1** | | **Model 2** | | **Model 3** | |
| --- | --- | --- | --- | --- | --- | --- |
|  | OR(95%CI) | *P*-value | OR(95%CI) | *P*-value | OR(95%CI) | *P*-value |
| TyG index^*^ | 1.299 (1.192-1.417) | <0.001 | 1.288 (1.181-1.406) | <0.001 | 1.370 (1.246-1.509) | <0.001 |
| TyG^※^ | | | | | | |
| Q1(< 8.912) | (Reference) |  | (Reference) |  | (Reference) |  |
| Q2(≥8.912) | 1.253 (1.108-1.417) | <0.001 | 1.239 (1.094-1.403) | <0.001 | 1.328 (1.161-1.520) | <0.001 |

**Notes:** #Stands for TyG index were continuous variable per 1 unit. ※Stands for the TyG continuous variables were divided into two groups based on the cut values obtained from the multivariate RCS regression analysis, with the lowest group used as the reference group. Model 1: unadjusted model; Model 2: adjusted for sex, age, laboratory parameters (albumin, serum potassium, serum sodium, creatinine, WBC, RBC, platelet); Model 3: adjusted for sex, age, laboratory parameters (albumin, serum potassium, serum sodium, creatinine, WBC, RBC, platelet) and comorbidities (congestive heart failure, chronic pulmonary disease, sepsis, myocardial infarction, liver disease, cerebrovascular disease, diabetes, renal disease, hypertension, malignant cancer);

**Abbreviations:** TyG, Triglyceride-glucose; OR, odds ratio; CI, confidence interval

# Supplementary Table 3. The association between various TyG index groups and risk of delirium in the ICU after excluding patients with sepsis (N=3,639).

|  | **Model 1** | | **Model 2** | | **Model 3** | |
| --- | --- | --- | --- | --- | --- | --- |
|  | OR(95%CI) | *P*-value | OR(95%CI) | *P*-value | OR(95%CI) | *P*-value |
| TyG index^*^ | 1.198 (1.089-1.318) | <0.001 | 1.195(1.085-1.316) | <0.001 | 1.293 (1.164-1.438) | <0.001 |
| TyG^*^ | | | | | | |
| Q1(< 8.912) | (Reference) |  | (Reference) |  | (Reference) |  |
| Q2(≥8.912) | 1.135 (0.995-1.295) | 0.059 | 1.130 (0.989-1.290) | 0.073 | 1.243 (1.077-1.435) | 0.003 |

**Notes:** #Stands for TyG index were continuous variable per 1 unit. ※Stands for the TyG continuous variables were divided into two groups based on the cut values obtained from the multivariate RCS regression analysis, with the lowest group used as the reference group. Model 1: unadjusted model; Model 2: adjusted for sex, age, laboratory parameters (albumin, serum potassium, serum sodium, creatinine, WBC, RBC, platelet); Model 3: adjusted for sex, age, laboratory parameters (albumin, serum potassium, serum sodium, creatinine, WBC, RBC, platelet) and comorbidities (congestive heart failure, chronic pulmonary disease, sepsis, myocardial infarction, liver disease, cerebrovascular disease, diabetes, renal disease, hypertension, malignant cancer);

**Abbreviations:** TyG, Triglyceride-glucose; OR, odds ratio; CI, confidence interval

# Supplementary Table 4. The association between various TyG index groups and risk of delirium in the ICU after excluding patients with diabetes (N=3,058).

|  | **Model 1** | | **Model 2** | | **Model 3** | |
| --- | --- | --- | --- | --- | --- | --- |
|  | OR(95%CI) | *P*-value | OR(95%CI) | *P*-value | OR(95%CI) | *P*-value |
| TyG index^*^ | 1.323 (1.187-1.476) | <0.001 | 1.305 (1.169-1.458) | <0.001 | 1.430 (1.273-1.609) | <0.001 |
| TyG^*^ | | | | | | |
| Q1(< 8.912) | (Reference) |  | (Reference) |  | (Reference) |  |
| Q2(≥8.912) | 1.334 (1.154-1.542) | <0.001 | 1.314 (1.135-1.522) | <0.001 | 1.475 (1.263-1.723) | <0.001 |

**Notes:** #Stands for TyG index were continuous variable per 1 unit. ※Stands for the TyG continuous variables were divided into two groups based on the cut values obtained from the multivariate RCS regression analysis, with the lowest group used as the reference group. Model 1: unadjusted model; Model 2: adjusted for sex, age, laboratory parameters (albumin, serum potassium, serum sodium, creatinine, WBC, RBC, platelet); Model 3: adjusted for sex, age, laboratory parameters (albumin, serum potassium, serum sodium, creatinine, WBC, RBC, platelet) and comorbidities (congestive heart failure, chronic pulmonary disease, sepsis, myocardial infarction, liver disease, cerebrovascular disease, diabetes, renal disease, hypertension, malignant cancer);

**Abbreviations:** TyG, Triglyceride-glucose; OR, odds ratio; CI, confidence interval

# Supplementary Table 5. The association between TyG index groups and risk of delirium in the ICU after propensity score matching (N=3,494).

|  | **Model 1** | | **Model 2** | | **Model 3** | |
| --- | --- | --- | --- | --- | --- | --- |
|  | OR(95%CI) | *P*-value | OR(95%CI) | *P*-value | OR(95%CI) | *P*-value |
| TyG index^*^ | 1.352 (1.228-1.490) | <0.001 | 1.343 (1.219-1.481) | <0.001 | 1.362 (1.231-1.508) | <0.001 |
| TyG^※^ | | | | | | |
| Q1(< 8.912) | (Reference) |  | (Reference) |  | (Reference) |  |
| Q2(≥8.912) | 1.353 (1.184-1.546) | <0.001 | 1.346 (1.177-1.539) | <0.001 | 1.368 (1.190-1.572) | <0.001 |

**Notes:** #Stands for TyG index were continuous variable per 1 unit. ※Stands for the TyG continuous variables were divided into two groups based on the cut values obtained from the multivariate RCS regression analysis, with the lowest group used as the reference group. Model 1: unadjusted model; Model 2: adjusted for sex, age, laboratory parameters (albumin, serum potassium, serum sodium, creatinine, WBC, RBC, platelet); Model 3: adjusted for sex, age, laboratory parameters (albumin, serum potassium, serum sodium, creatinine, WBC, RBC, platelet) and comorbidities (congestive heart failure, chronic pulmonary disease, sepsis, myocardial infarction, liver disease, cerebrovascular disease, diabetes, renal disease, hypertension, malignant cancer);

**Abbreviations:** TyG, Triglyceride-glucose; OR, odds ratio; CI, confidence interval

# Supplementary Table 6. The baseline clinical characteristics of the patients after propensity score matching in both TyG groups were analyzed.

| **Categories** | **Total** | **Q1(< 8.912)** | **Q2(≥ 8.912)** | ***P* value** |
| --- | --- | --- | --- | --- |
| N | 3,494 | 1,747 | 1,747 |  |
| **Demographic** |  |  |  |  |
| Age, years, | 76 (70,83) | 76 (70,83) | 76 (70,83) | 0.79 |
| Sex, n(%) |  |  |  | 0.892 |
| Male | 1,879 (53.7) | 937 (53.6) | 941 (53.9) |  |
| Female | 1,616 (46.3) | 910 (46.4) | 806 (46.1) |  |
| Ethnicity, n(%) |  |  |  | 0.13 |
| White | 2,379 (68.1) | 1,116 (66.7) | 1,213 (69.4) |  |
| Other | 1,115 (31.9) | 581 (33.3) | 534 (30.6) |  |
| **Vital signs** |  |  |  |  |
| Temperature, °C | 36.7 (36.4,37.1) | 36.7 (36.4,37.0) | 36.7 (36.4,37.1) | 0.014 |
| Heart rate, beats/minute | 86 (73,100) | 84 (73,99) | 87 (74,101) | 0.002 |
| Mean blood pressure, mmHg | 84 (72,97) | 83 (71,96) | 84 (72,97) | 0.110 |
| Respiratory rate, beats/minute | 19 (16,24) | 19 (16,23) | 19 (16,24) | 0.007 |
| **Laboratory tests** |  |  |  |  |
| Albumin, g/dL | 3.4 (2.8,3.9) | 3.4 (2.8,3.9) | 3.3 (2.8,3.9) | 0.412 |
| BUN, mg/dL | 22 (16,35) | 22 (15,34) | 23 (16,36) | 0.046 |
| Creatinine, mg/dL | 1.1 (0.8,1.5) | 1 (0.8,1.5) | 1.1 (0.8,1.5) | 0.034 |
| Serum sodium, mEq/L | 139 (136,142) | 139 (136,142) | 139 (136,141) | 0.378 |
| Serum potassium, mEq/L | 4.1 (3.8,4.6) | 4.1 (3.8,4.6) | 4.2 (3.8,4.6) | 0.415 |
| INR | 1.2 (1.1,1.5) | 1.3 (1.1,1.5) | 1.2 (1.1,1.5) | 0.011 |
| WBC, 10^9^/L | 10.8 (7.8,14.9) | 10.3 (7.5,14.4) | 11.2 (8.5,15.2) | < 0.001 |
| RBC, 10^9^/L | 3.6 (3.1,4.2) | 3.6 (3.1,4.2) | 3.7 (3.1,4.2) | 0.429 |
| Platelet, 10^9^/L | 205 (150,269) | 206 (150,265.5) | 204 (149,274) | 0.944 |
| Triglycerides, mg/dL | 111 (80,156) | 82 (64,102) | 153 (120,206) | < 0.001 |
| Glucose, mg/dL | 132 (106,169) | 113 (96,137) | 156 (126,196) | < 0.001 |
| **Comorbidities** |  |  |  |  |
| Sepsis, n(%) |  |  |  | 0.718 |
| No | 2,687 (76.9) | 1,339 (76.6) | 1,348 (77.2) |  |
| Yes | 807 (23.1) | 408 (23.4) | 399 (22.8) |  |
| Myocardial infarction, n(%) |  |  |  | 0.734 |
| No | 2,517 (72) | 1,254 (71.8) | 1,263 (72.3) |  |
| Yes | 977 (28) | 493 (28.2) | 484 (27.7) |  |
| Cerebrovascular disease, n(%) |  |  |  | 0.26 |
| No | 2,230 (63.8) | 1,131 (64.7) | 1,099 (62.9) |  |
| Yes | 1,264 (36.2) | 616 (35.3) | 648 (37.1) |  |
| Congestive heart failure, n(%) |  |  |  | 0.943 |
| No | 2,284 (65.4) | 1,143 (65.4) | 1,141 (65.3) |  |
| Yes | 1,210 (34.6) | 604 (34.6) | 606 (34.7) |  |
| Chronic pulmonary disease, n(%) |  |  |  | 0.97 |
| No | 2,505 (71.7) | 1,253 (71.7) | 1,252 (71.7) |  |
| Yes | 989 (28.3) | 494 (28.3) | 495 (28.3) |  |
| Diabetes, n(%) |  |  |  | 0.245 |
| No | 2,384 (68.2) | 1,208 (69.1) | 1,176 (67.3) |  |
| Yes | 1,110 (31.8) | 539 (30.9) | 571 (32.7) |  |
| Renal disease, n(%) |  |  |  | 0.37 |
| No | 2,617 (74.9) | 1,297 (74.2) | 1,320 (75.6) |  |
| Yes | 877 (25.1) | 450 (25.8) | 427 (24.4) |  |
| Malignant cancer, n(%) |  |  |  | 0.46 |
| No | 2,936 (84) | 1,476 (84.5) | 1,460 (83.6) |  |
| Yes | 558 (16) | 271 (15.5) | 287 (16.4) |  |
| Liver disease, n(%) |  |  |  | 0.955 |
| No | 3,141 (89.9) | 1,570 (89.9) | 1,571 (89.9) |  |
| Yes | 353 (10.1) | 177 (10.1) | 176 (10.1) |  |
| Hypertension, n(%) |  |  |  | 0.361 |
| No | 1,759 (50.3) | 893 (51.1) | 866 (49.6) |  |
| Yes | 1,735 (49.7) | 854 (48.9) | 881 (50.4) |  |
| **Events** |  |  |  |  |
| RRT treatment, n(%) |  |  |  | 0.107 |
| No | 3,160 (90.4) | 1,594 (91.2) | 1,566 (89.6) |  |
| Yes | 334 (9.6) | 153 (8.8) | 181 (10.4) |  |
| MV, n(%) |  |  |  | < 0.001 |
| No | 1,771 (50.7) | 1,006 (57.6) | 765 (43.8) |  |
| Yes | 1,723 (49.3) | 741 (42.4) | 982 (56.2) |  |
| ICU death, n(%) |  |  |  | 0.708 |
| Survivor | 3,099 (88.7) | 1,553 (88.9) | 1,546 (88.5) |  |
| Non-survivor | 395 (11.3) | 194 (11.1) | 201 (11.5) |  |

**Abbreviations:** MIMIC-IV, Medical Information Mart for Intensive Care IV; eICU-CRD, eICU Collaborative Research Database; WBC, White cell count; RBC, Red cell count; RRT, Renal replacement therapy; MV, Mechanical ventilation; TyG, Triglyceride-glucose; ICU, Intensive care unit.

# Supplementary Table 7. Subgroup analysis for the association of TyG index with the risk of delirium in ICU patients aged 65 and above.

|  | **Case/Total** | **Q1(< 8.912)** | | **Q2(≥8.912)** | | ***P* for interaction** |
| --- | --- | --- | --- | --- | --- | --- |
|  |  | **OR(95%CI)** | ***P*** | **OR(95%CI)** | ***P*** |  |
| Sex |  |  |  |  |  | 0.570 |
| Male | 1,163/2,506 | (ref) | (ref) | 1.332 (1.122-1.580) | <0.001 |  |
| Female | 1,002/2,143 | (ref) | (ref) | 1.325 (1.100-1.600) | 0.003 |  |
| Age(years) |  |  |  |  |  | 0.671 |
| < 80 | 1,355/2,937 | (ref) | (ref) | 1.272 (1.087-1.490) | 0.003 |  |
| ≥80 | 810/1,712 | (ref) | (ref) | 1.400 (1.136-1.730) | 0.002 |  |
| Ethnicity |  |  |  |  |  | 0.586 |
| White | 1,795/3,168 | (ref) | (ref) | 1.315 (1.129-1.530) | <0.001 |  |
| Other | 689/1,481 | (ref) | (ref) | 1.342 (1.072-1.680) | 0.011 |  |
| Congestive heart failure |  |  |  |  |  | 0.770 |
| No | 1,405/2,978 | (ref) | (ref) | 1.292 (1.106-1.510) | <0.001 |  |
| Yes | 760/1,671 | (ref) | (ref) | 1.380 (1.110-1.720) | 0.004 |  |
| Chronic pulmonary disease |  |  |  |  |  | 0.726 |
| No | 1,549/3,356 | (ref) | (ref) | 1.287 (1.110-1.490) | <0.001 |  |
| Yes | 616/1,293 | (ref) | (ref) | 1.424 (1.119-1.810) | 0.004 |  |
| Hypertension |  |  |  |  |  | 0.910 |
| No | 1,116/2,310 | (ref) | (ref) | 1.313 (1.098-1.570) | 0.003 |  |
| Yes | 1,049/2,339 | (ref) | (ref) | 1.340 (1.121-1.600) | <0.001 |  |
| Sepsis |  |  |  |  |  | 0.322 |
| No | 1,549/3,639 | (ref) | (ref) | 1.243 (1.077-1.440) | 0.003 |  |
| Yes | 616/1,010 | (ref) | (ref) | 1.559 (1.193-2.040) | <0.001 |  |
| Renal disease |  |  |  |  |  | 0087 |
| No | 1,570/3,446 | (ref) | (ref) | 1.315 (1.137-1.520) | <0.001 |  |
| Yes | 595/1,203 | (ref) | (ref) | 1.273 (0.992-1.630) | 0.058 |  |

**Abbreviations:** OR, odds ratio; CI, confidence interval

# Supplementary Figure 1. Kaplan-Meier analysis showed the cumulative incidence of mortality at 30 days, 90 days, and 360 days after delirium in ICU patients aged 65 and above with TyG index.


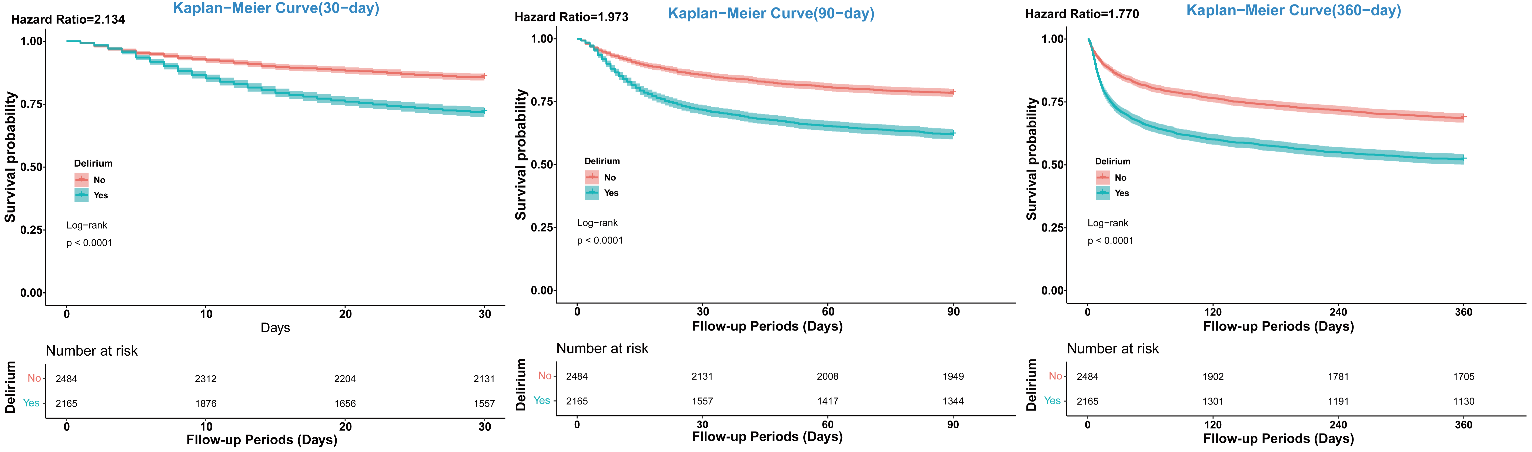

Supplement: Supplementary file 1 — Supplementary Material 1 [file 12877_2023_4420_MOESM1_ESM.docx]
